# A Symmetric Encoder–Decoder Network with Enhanced Group–Shuffle Modules for Robust Lung Nodule Detection in CT Scans

**DOI:** 10.3390/biomimetics11020092

**Published:** 2026-02-01

**Authors:** Mohammad A. Thanoon, Siti Raihanah Abdani, Ahmad Asrul Ibrahim, Asraf Mohamed Moubark, Nor Azwan Mohamed Kamari, Muhammad Ammirrul Atiqi Mohd Zainuri, Mohd Hairi Mohd Zaman, Mohd Asyraf Zulkifley

**Affiliations:** 1 Department of Electrical, Electronic and Systems Engineering, Faculty of Engineering and Built Environment, Universiti Kebangsaan Malaysia (UKM), Bangi 43600, Selangor, Malaysia; p119159@siswa.ukm.edu.my (M.A.T.);; 2 System and Control Engineering Department, College of Electronics Engineering, Ninevah University, Mosul 41002, Iraq; 3 School of Computing Sciences, College of Computing, Informatics and Mathematics, Universiti Teknologi MARA, Shah Alam 40450, Selangor, Malaysia

**Keywords:** lung cancer detection, CT image segmentation, group–shuffle hybrid model, deep learning, automated disease screening

## Abstract

Lung cancer is considered to be a significant cause of death in the world, and the timely identification of nodules in the lungs in CT scans is very important to enhance the prognosis of patients. Although the state of the art of nodule delineation using deep learning-based segmentation models was achieved, major problems, including high feature diversity, low spatial discrimination, and overfitting of the models, require stronger feature-processing approaches. This research explores an enhanced symmetric encoder–decoder segmentation network known as the Improved Group–Shuffle Module (IGSM) to overcome these shortcomings. The most important feature of the proposed method is the IGSM, which hierarchically divides feature maps into a few groups, then transforms them independently, and then randomly switches channels between groups to increase inter-group interaction of features and diversity. This IGSM method is inspired by human brain functions, which are processed in specialized cortex areas, which are mimicked in this work through small-group feature processing. Channel shuffling is designed based on inter-modular communication in the human brain through coherent information sharing among the small groups of cortices. Through this mechanism, the model is much better at capturing discriminative spatial and contextual patterns, especially on complex and subtle nodule structures. The IGSM configurations have been optimized, specifically, the placement of the modules, grouping size, and shuffle permutation strategies. The proposed model’s performance is then compared with the benchmarked models, like U-Net and DeepLab, with various performance indicators such as mean Intersection over Union (mIoU), Dice Score, Accuracy, Sensitivity, and Specificity. The simulation results proved the superiority of the IGSM-enhanced model with the mIoU of 0.7735, the Dice Score of 0.9665, and the Accuracy of 0.9873. The addition of the group and shuffle module not only enhances the discrimination between the nodules and their background, but it also improves the ability to generalize over a variety of nodules’ morphology, thus producing a reliable tool for automated detection of lung cancer.

## 1. Introduction

Lung cancer is a leading global type of cancer that generates the largest number of deaths and morbidity among all other cancers. The efficacy of lung cancer treatment relies on the early detection and accurate diagnosis for better therapeutic effects [1]. Medical imaging examination utilizes deep learning algorithms and image processing techniques for carrying out automated lung cancer detection and diagnosis in CT and X-ray images [2]. The conventional methods of lung cancer classification are not robust because tumors have different shapes and various grain sizes, especially when they move within the body of a patient. The proposed solution of this study significantly improves segmentation results with the aid of data shuffle combined with a series of segmentation techniques. Furthermore, better network generalization capability is achieved using segmentation methods with data shuffling to enable the accurate separation and identification of lung nodules [3].

CT scans are used in medical imaging technologies to detect lung cancer because these scans generate clear pictures and detailed structures of lung tissues while offering maximum image resolution. The precise segmentation of medical pathologies is normally difficult because of complex lung tissue structures and heterogeneous tumors that can create substantial visual obstacles [4]. Previously, Support Vector Machines (SVMs) and Random Forest models have been used by medical professionals to screen and diagnose lung cancer, but due to their manual feature extraction process, detection rates are rarely high. Hence, the deep learning approach, through Convolutional Neural Networks (CNNs), is explored to better extract unique features and allow them to perform better than the conventional approaches [5]. Furthermore, deep learning models with data shuffling capability improve segmentation performance to produce better generalization and lower overfitting effects. Moreover, random dataset distribution during the model fitting process through a data shuffling strategy stops the model from developing pattern biases in sequential data. This data shuffling process was inspired by the inter-modular communication in the human brain, which executes coherent information sharing among several small groups of cortices. By sharing and merging the data among the cortices, the learning process utilizes and excites various paths of neuron learning, not limited to the dominant paths. Data shuffling is particularly beneficial for processing medical images because it produces models that are independent of image acquisition variations and patient-specific variations [6]. This shuffling requires grouping of smaller sets of feature maps, whereby the information is compartmentalized to reduce the likelihood of dominant paths of feature learning. This learning process is synonymous with human brain operations, where several excitations of various parts of the cortex will usually lead to better feature or pattern extraction.

The shuffling technique is further supplemented by the diversity of training samples through image processing-based rotation and flipping operators, as well as contrast variations. Such a process makes the model more discriminative and able to learn features shared across imaging variations. In addition, inclusion of data shuffling methods in the training pipeline allows for more robust generalization of diagnostic models towards never-before-seen lung cancer patients, and thus increases the reliability of diagnostics [7]. In this work, the goal of the segmentation task is to extract regions of interest (ROI) in lung images, thereby making feature extraction accurate and avoiding unnecessary data. Various advanced segmentation methods, including threshold-based approaches, region-growing techniques, as well as deep learning-based methods like U-Net and Mask R-CNN, have been proposed to detect and segment lung nodules in scans [8]. In general, medical image segmentation helps doctors to interpret visual information better and decrease the likelihood of misidentification of positive cases [9].

Usually, deep learning-based segmentation techniques implemented encoder–decoder designs for obtaining hierarchical features prior to achieving precise lung nodule segmentations. For example, many medical image segmentation applications adopt a U-Net-like architecture as the preferred approach because of its capability in the retrieval of spatial and contextual information. It allows for disease-specific region analysis by combining segmentation and classification tasks, which improves its ability to detect lung cancer [10]. Current deep learning segmentation advancements alone are not enough to resolve the complete range of problems involving lung cancer detection. These models reported decreased performance levels, mainly because of different feature challenges in the datasets. The different dimensions and positions of cancer nodules create issues of non-uniform segmentation outputs. Sometimes, the learning model produces skewed results due to the fact that benign cases outnumber malignant cases in most datasets [11]. The processing methods should also be considered a critical performance benchmark for lung cancer detection. Furthermore, the processing requirements of deep learning segmentation models require more computational demand compared to current standard clinical processing systems, which prevents successful applications of real-time deployment. Methods for model-pruning, along with network optimization, enhance the performance efficiency of systems without affecting the accuracy results [12].

This work investigates the advantages of employing various advanced segmentation modules for lung cancer analysis. The developed automated detection system aims to facilitate physicians by offering precise diagnosis help when detecting early-stage lung cancer. In the next section, this paper discusses related studies, followed by a methodology section, discussions of the simulation results, and conclusions. Primarily, this paper aims to enhance lung cancer detection model reliability and effectiveness through embedding advanced modules like group and shuffle units.

## 2. Background

### 2.1. Related Works

Zhang et al. [13] adapted the ResNet architecture to create a modification for the NSCLC inoperable patient’s tumor volume segmentation. Their framework employed an encoder–decoder framework that resembles the U-Net to obtain deep features from CT scans by using ResNet34 as its backbone. This network processed information at different spatial scales by uniting deep semantic characteristics with basic features to boost its segmentation precision level. The research group obtained strong results from their algorithm, which yielded a 0.73 Dice Similarity Coefficient while showing better performance than the standard U-Net architecture. The system requires expansion of its training data volume and resolution capabilities to properly segment small tumors and tumors that are located near delicate structures like the mediastinum.

Mishra and Gangwar [14] conducted research using three separate datasets to evaluate deep learning models for detecting lung cancer by segmenting the nodules of interest. A U-Net CNN model obtained an initial DSC of 67.8% through its application. The second CNN built after the initial model worked to reduce false positives until it reached a validation accuracy of 84.4%. The combined models demonstrated a detection performance with 0.75 sensitivity, demonstrating their capacity to find true positives and control false positives. Researchers demonstrated that multiple interconnected models deliver superior outcomes for detecting lung cancer correctly.

Angeline et al. [15] designed a convolutional neural network (CNN) for lung nodule segmentation. This deep learning model segmented CT scan ROIs containing potential nodules, after which it utilized multiple convolutional layers to determine pixel classification, either as nodule tissues, vessel structures, or background tissues. Zero-center normalization, together with a SoftMax layer, operated as the classification method for pixels in the model. This research illustrated that deep learning models work effectively for detecting and segmenting lung nodules, which leads to early lung cancer detection.

Meraj et al. [16] investigated deep learning strategies for detecting lung cancer malignancy through their research. VGG-16 operated on CT scans to find lung nodules while classifying their status between benign, unsure, and malignant. The VGG-16 model, which uses deep layers with filters, attained an 78.87% accuracy rate in tumor type classification. The research findings proved that deep learning networks, specifically VGG-16, can establish strong diagnostic solutions for lung cancer, which efficiently identify multiple tumor types.

A deep learning tool for lung nodule detection received performance testing against findings from radiologists during manual examinations in [17]. The authors implemented multiple CNNs with a U-Net design to scan CT images for potential nodule candidates, then used FROC evaluation to assess their results. The DL-CAD system achieved a sensitivity of 90.1% compared to double readings performed by radiologists, which returned 76.0% sensitivity. The system proved better than human radiologists by identifying nodules that escaped human detection while performing with steady accuracy for lung cancer detection.

Furthermore, Salama et al. [18] developed a framework to generate synthetic chest X-ray (CXR) images combined with tumors of different sizes and positions through generative modeling. The proposed method managed to balance the class distribution throughout the dataset by addressing the shortage of labeled CXR images. ResNet50 demonstrated 98.91% detection accuracy through its training on synthetic medical images.

On the other hand, the work in [19] developed a CNN model that incorporated a bottleneck design to lower feature map resolution. The researchers applied chest radiographs alongside their inverted images as a method of data enhancement in their study. The model displayed a mean sensitivity of 0.73 but faced difficulties locating tumors located at the apices, as well as tumors near essential anatomical structures. These findings demonstrated the problem of identifying tumors in blind spots and normal regions when tumor sizes were large, which requires additional work to improve model development.

Besides that, Gunasekaran [20] applied YOLOv5 for object detection in lung cancer identification projects. The implemented model possessed three main modules, which included feature extraction, followed by pyramid feature generation and detection prediction of cancer areas. The detection methodology produced successful results that demonstrated 94% sensitivity, together with 90.5% specificity and perfect 100% precision. This research showed that YOLOv5 proved capable of performing both precise lung cancer detection and excellent identification of malignant regions in chest X-ray images.

Researchers in [21] combined MobileNetV2 and U-Net to form a hybrid architecture for the segmentation of lung tumors in CT. In the U-Net’s backbone, they used the MobileNetV2 model to serve as the encoder, which facilitated the rapid processing of complex feature extraction work. This hybrid model showed its superior accuracy by achieving a Dice score of 0.8793, a recall of 0.8602, and a precision of 0.93 in identifying lung tumors. The work proves that further model optimization with diverse datasets would improve the accuracy of segmentation.

In [22], the authors employed various GAN structures for image translation, transforming original lung CT scans to segmented outcomes. Segmentation was performed at the generator since it generated a series of distinct CT input scans, and the discriminator focused on overcoming the limitation of segmentation accuracy. The use of GANs with this generative adversarial structure achieved accurate segmentation of realistic lung images, demonstrating the power and potential to improve medical image segmentation quality using GANs.

Finally, Gao et al. [23] proposed a growth prediction pipeline with WGAN for the prediction of lung nodule change in CT follow-up scans. The model performed well in matching nodule images within 1 year of each other, with 0.827 test AUC for future growth prediction on nodules. The model provided accurate predictions of the levels of lung nodule progression, which is even beyond actual follow-up image accuracy and is a big step forward in the early diagnosis and risk assessment of lung cancer.

### 2.2. Lung Cancer Segmentation

Lung nodule segmentation achieves paramount importance in medical diagnostics and treatment decisions for lung cancer because it enables early-stage detection while measuring cancer malignancy and monitoring treatment responses [24]. Manual segmentation of lung nodules was the standard practice until recently, when CAD systems provided supplementary assistance. These methods depend on human input with significant variations between different radiologists who conducted the assessments [25].

Advanced imaging devices like X-rays and CT scans, together with MRIs, cannot eliminate the risk of ignoring nodules because these nodules can remain undetected, particularly when they are tiny or situated within hard-to-spot lung regions [26].

Computer-aided detection systems prove their value through their ability to solve different system issues. CAD systems feature two detection capabilities for clinical opinion generation and cancer detection of obscured nodules that result in better early diagnosis. Lung nodule detection through standard screening yields successful results in only two-thirds of cases because human examiners overlook one-third of such nodules [27].

Because CT scans have been progressively increasing in quantity, yet trained radiologists remain scarce, the medical field now faces an excessive workload for professionals who handle these scans. Excessive workload and fatigue, together with stress conditions, make errors more likely and increase the chance of diagnostic omissions [28]. The manual examination of small lung lesions and spots becomes challenging because human vision has limitations when detecting tiny elements and when they appear in problematic viewing positions or have sizes below the boundaries. Medical professionals reveal that human visual capacities have their limitations, so advanced computer models become essential for spotting faint abnormalities [29].

Medical images often present difficulties because of variations that stem from different scanner models, together with imaging settings, as well as patient positioning. Different types of variations affect the performance of segmentation algorithms, which results in irregular segmentation outcomes [30]. Medical images sometimes include noise or artifacts that prevent nodules from being visible while generating false positive results, primarily in cases of poor image quality [31].

The segmentation process becomes complex because lung nodules demonstrate natural variations between patients. Patient lung nodules show extensive differences across individuals because they exhibit differences in size and shape as well as texture characteristics [32]. Lung nodule appearance changes during the same patient period, which brings difficulties to both identification and tracking methods [33]. The evaluation of lung tissue in scans becomes more complicated because patient age, along with existing medical conditions and smoking habits, affects tissue appearance, which makes it difficult to separate nodules from other lung elements [34].

The accurate segmentation process faces another barrier because of the nodular patterns. The detection and segmentation of large solid nodules is straightforward, yet small nodules near blood vessels or lung walls prove extremely hard to notice. Early lung cancer detection requires special to small nodules since they represent the initial stage of cancerous growth [35]. The partial volume effect represents one of the main difficulties with small nodules because the nodule appears across the boundaries of multiple scan images. The detection of nodules with ground-glass opacity (GGO) stands as one of the most challenging tasks when scanning the lungs. The lighter, less dense appearance of these nodules creates difficulties in distinguishing them from normal lungs on imaging scans [36].

**Figure 1:**
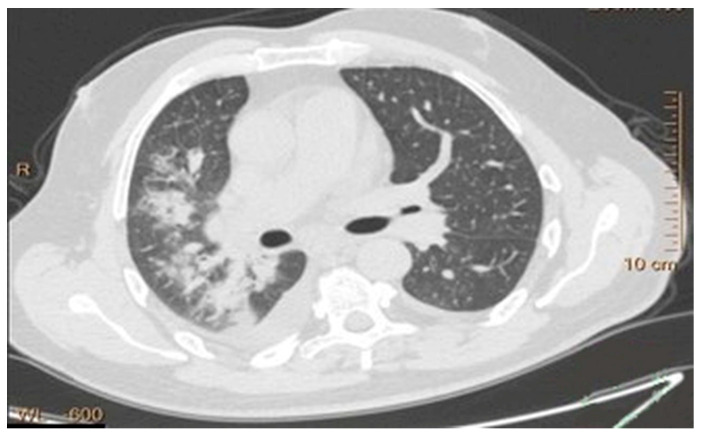
Lung cancer nodules in CT scans.

Lung cancer diagnosis, as shown in Figure 1, becomes more difficult due to its diverse nature as well as the range of growth patterns and visual characteristics across various cancer types. Several distinct types of lung cancer may present themselves differently within a single imaging examination [37]. The dominant subtype usually takes priority in diagnosis procedures performed by radiologists, yet this practice omits critical details from less obvious subtypes. Acute and complete subtype identification and characterization enable medical professionals to select optimal treatment solutions. The correct determination of benign versus malignant status depends heavily on evaluating size, growth rate, and density characteristics in nodules [24].

The survival rates for lung cancer improve significantly when patients receive early diagnosis because doctors can treat cancers discovered at this stage better and achieve better results. Timely medical response leads to significantly improved results, which lower patient death rates alongside hospitalization risks. Early detection needs make CAD systems exist as essential components for medical diagnosis [38]. CAD systems serve radiologists in detecting nodules while simultaneously building an extra evaluation system to stop potential nodule escapes. The technology proves beneficial for healthcare providers who lack expertise in lung cancer diagnosis, along with trainees, because the system provides them with guidance that minimizes diagnostic errors [39].

Higher levels of accuracy remain elusive, as the systems continue to become more complex. The goal of the medical community is to develop CAD systems capable of near-perfect segmentation and detection tasks for aiding in lung cancer diagnosis. For life-threatening diagnostic scenarios, CAD systems need to be more accurate with fewer false alarms and human interference [27]. The diagnosis of lung cancer will evolve with the improved utilization of AI-based tools combined with human physician capabilities to produce more rapid and accurate analytic results. Continuous development on enabling systems to work at peak performance to aid medical professionals and increase patient outcomes is needed [40].

## 3. Methodology

This work presents a novel symmetric encoder–decoder segmentation framework that is specifically designed for efficient lung tumor delineation in computed tomography images. It is built from scratch as a custom baseline model, with both encoder and decoder having the same number of stages that maintain structural symmetry. Core to this work is the embedding of an enhanced Group–Shuffle module within selected blocks of the encoder and decoder to capture efficient channel interaction, strengthen feature diversity, and improve multi-level representation learning. This work follows the design philosophy adopted in recent lightweight convolutional systems developed within our research laboratory, in which group interactions and channel-shuffling operations have demonstrated superior efficiency-accuracy trade-offs.

### 3.1. Baseline Encoder–Decoder

**Figure 2:**
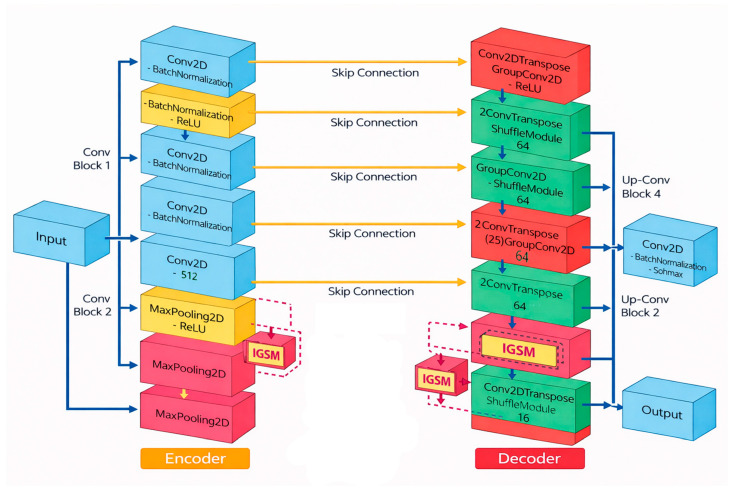
CNN-based encoder–decoder model with Group–Shuffle module.

The proposed segmentation approach’s encoder–decoder skeleton is established with the use of a carefully designed Symmetric Encoder–Decoder Network, as shown in Figure 2. Encoder layers allow for a successive reduction in image resolution with the use of a combination of convolutional layers as well as down-sampling layers, so that the successive increase in depth helps in the better abstraction of cyclically increasing contextual information. Each stage in the convolutional layers in the encoder is designed with sequential convolution, normalization, and activation layers, so that the stable back-propagation of gradients is achieved along with efficient feature abstraction. In addition, skip-connect layers ensure that there is preservation of important information associated with image localization during the encoding process, as shallow feature maps with rich edge information are transmitted directly from the encoder layers and corresponding decoder layers. Unlike traditional encoder–decoder networks, which were based essentially on conventional convolutional layers, the proposed design explicitly facilitates better feature mixing, abstraction, and reuse, laying down the groundwork for seamless addition of the Group–Shuffle (GS) module.

### 3.2. Improved Group–Shuffle Module

To address the disadvantages of isolated channel groups, as well as a lack of channel communications, the enhanced GS module is integrated throughout strategic layers of the network. Inside every GS module, the feature maps will be split into multiple channel groups, with transformations applied independently, followed by a channel shuffle layer that helps guarantee propagation of information across channel groups. This helps prevent representative knowledge stagnation, as well as provides enhanced feature interaction capabilities.

In its enhanced form, the GS module brings forth multiple improvements compared with traditional GS modules, such as adaptive group sizes that depend on layer depth, cross-group fusion strategies that fuse information prior to, as well as subsequent to, channel shuffling, and a spatially informed channel shuffle pattern that helps guarantee spatial coherence, along with channel diversity. These improvements provide better feature expressiveness capabilities, as well as allow for reduced computational costs, with even more effective complex tumor boundary definitions. This is based on principles validated within multiple GS-based studies conducted within our group, which emphasized group convolution, channel shuffling, and hybrid mixing. Such principles were shown to be effective across multiple applications within the field of medical imaging. By integrating the GS module within both the network decoder and encoder, this provides capabilities for improved feature propagation facilitated through multiple abstraction levels, yet remains computationally efficient.

Let $X\in R^{C\times H\times W}$ denote the input feature map with *C* channels and spatial dimensions *H* × *W*. The Improved Group–Shuffle Module (IGSM) produces an output feature map $Y\in R^{C\times H\times W}$ through grouped processing, shuffling, and residual fusion.

The input channels are first divided into G groups of equal size, where G is a hyperparameter determined by the layer depth (typically G∈{4,8,16} in our architecture). The partitioning operation $\rho_{g}$ for group g (g=1,2,…,G) is defined as:

(1) $$X_{g}\;=\;\rho_{g}(X)\in R^{(C/C)\;\times H\;\times \;W}$$

where $X_{g}$ represents the feature sub-map belonging to group *g.*

Each group undergoes an independent lightweight transformation $\tau_{g}$ to learn specialized feature representations. This transformation comprises a sequence of operations:

(2) $$Z_{g}\;=\;\tau_{g}\left(X_{g}\right)\;=\;B\;N\left(ReLU\left({Conv}_{3\;\times \;3}\left(X_{g}\right)\right)\right)$$

where ${Conv}_{3\times 3}$ denotes a standard 3 × 3 convolution applied within the group, *BN* is Batch Normalization, and *ReLU* is the Rectified Linear Unit activation. The convolution uses a stride of 1 and padding of 1 to preserve spatial dimensions. The group-specific processing mimics the modular, specialized processing observed in cortical regions of the human brain.

To facilitate cross-group information exchange—inspired by inter-modular neural communication—the transformed group features $\left\{Z_{1},Z_{2},Z_{G}\right\}$ are concatenated along the channel dimension and then subjected to a channel shuffle operation S. The concatenation yields:

(3) $$Z_{cat}\;=\;Concat\;\left(Z_{1},Z_{2},,\;Z_{G}\right)\in R^{C\times H\times W}$$

The channel shuffle operation S systematically permutes the channels of $Z_{cat}$ to ensure information flow between groups that were processed independently. Formally, if we reshape the channel dimension *C* into a matrix of size (G,C/G), transposing this matrix and flattening it back implements the shuffle:

(4) $$Z_{shuffled}\;=\;\left(S|Z_{cat}\right)\;=\;Flatten\;\left(Transpose\;\left(Reshape\;\left(Z_{cat},\;\left(G,\frac{c}{G},H,W\right)\right)\right)\right)$$

where Reshape reorganizes the tensor, Transpose swaps the first two dimensions (group and intra-group channel), and Flatten restores the original channel dimension. This operation ensures that each channel in the subsequent layer receives input from a different subset of groups in the previous layer, promoting feature diversity. The shuffled features pass through a final pointwise convolution (1 × 1 *Conv*) to fuse information across all channels:

(5) $$Y^{\prime }\;={\;Conv}_{1\;\times \;1}(Z_{shuffled})$$

Finally, to preserve gradient flow and enable identity mapping, a residual connection is added:

(6) $$Y\;=\;Y^{\prime }\;+\;X$$

This formulation ensures that the IGSM enhances feature representation diversity while maintaining computational efficiency, as group convolutions reduce parameters by a factor of G compared to standard convolutions, and the shuffle operation introduces negligible computational overhead.

### 3.3. Hierarchical Features Fusion

**Figure 3:**
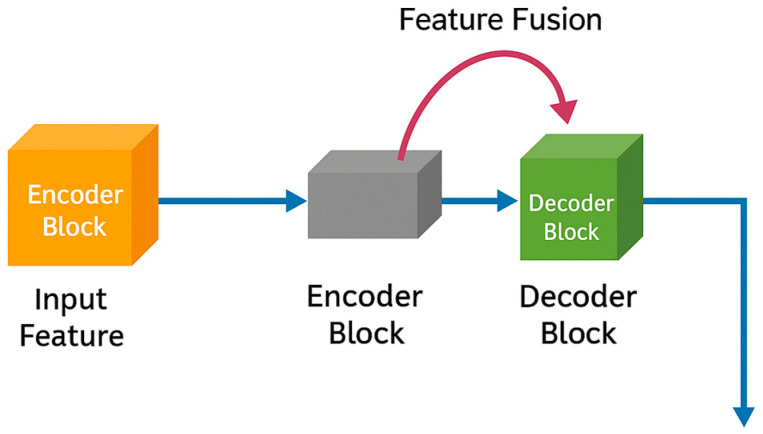
Hierarchical features fusion layers.

An essential aspect of the proposed architecture is the use of a hierarchical fusion method in Figure 3 that incorporates encoder and decoder feature maps, enabling the model to achieve accurate spatial reconstruction. During this process, the use of skip-connect modules becomes essential as the fine details may attenuate as the resolution contracts, with the proposed framework conducting fusion on GS-enhanced encoder feature maps with their associated decoder modules via a concatenation procedure that is further intensified with refinement convolutions. This will allow the network, despite its focus on deeper feature representations, to leverage low-level as well as high-level feature maps for reconstructive purposes.

This is particularly helpful as tumor boundaries, exhibiting subtle as well as irregular variations, may often pose difficulties within CT images. By effectively incorporating knowledge spanning multiple scales, from textural representations in early stages to higher-level semantics, the proposed architecture neither remains dependent on deeper feature representations nor neglects the detection of lesions that may appear as irregularities.

### 3.4. Decoder Reconstruction and Final Prediction

**Figure 4:**
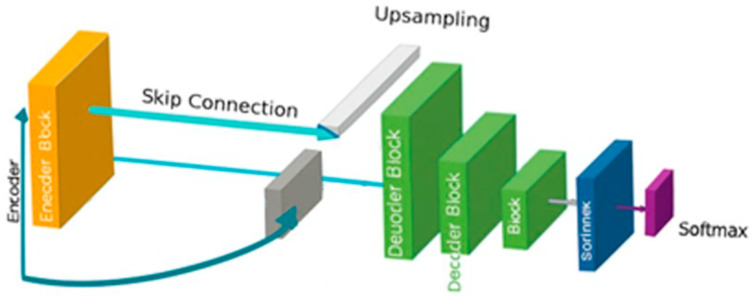
Decoder reconstruction layers.

Unlike the encoder, the decoder is as complex as the encoder, with inverse operations that focus on the restoration of spatial dimensions. Each up-sampling block is driven by feature fusion as indicated in Figure 4, whereby GS-enhanced encoder features fuse, allowing the decoder to focus on tumor boundary reconstruction with higher resolution. Transposed convolution up-sampling or traditional up-sampling through interpolation is necessary for smooth transitions of spatial dimensions. Finally, convolutional refinements, achieved through convolutional layers, solidify the enhanced feature information. Deep into the decoder, the incorporation of fine details of the encoder with abstract representations of deeper hierarchies culminates in a segmentation map with global as well as local representations of the tumor.

Finally, the last layer of the decoder, designed as the prediction layer, utilizes a 1 × 1 convolutional layer with a SoftMax activation that provides pixel-wise predictions of tumor or non-tumor classes. Finally, the output segmentation mask provides a comprehensive overview of feature processing hierarchies, as well as GS-driven feature diversification, as seen in the proposed framework. It is worth mentioning that this proposed model is entirely self-made, being exclusive for CT lung image segmentation purposes, with no adjustments or derivatives from any existing framework.

Experimental Baseline and Benchmarking Framework. Computational capabilities, as a significant element within a company, are examined through the application of multiple benchmarking models to fulfill the essential function of assisting companies in accomplishing their objectives. As an important component in a firm, the capabilities of computation are studied by implementing various models in benchmarking as a means of completing the necessary role of helping firms achieve their goals.

To fully achieve the validation of the effectiveness of the introduced symmetric encoder–decoder framework with IGSM, we compare it with five popular segmentation models: U-Net, DeepLabV2, PSPNet, DABNet, and HRNet. The models were chosen to cover a range of architectural philosophies, as well as reflect major progress in medical and general image segmentation:

1. U-Net: An iconic, massively used baseline in semantic medical image segmentation, which is characterized by an encoder–decoder format with skip connections. It is added in order to create an underlying bonus reference.
2. DeepLabV2 Ref. [26]: Represents the family of models that applies to the multi-scale context aggregation option without significant drops in resolution. It compares the ability of our model with the methods that are oriented towards contextual feature extraction.
3. PSPNet: Adds two types of pooling modules, so-called pyramid pooling, to narrow down the context on the global and local levels. It forms a reference point to models that place superior emphasis on the integration of contextual prior in multi-regions, with an important role to objects of different scales.
4. DABNet: This is a lightweight model that has dual variables (channel and spatial) to increase the feature discrimination. It is selected to test our model with respect to the recent lightweight, efficiency-oriented designs.
5. HRNet: Uses high-resolution feature representations in the entire network, which is not the case with typical encoder–decoders, which down-sample over and over again. It compares the quality of our model when it comes to maintaining spatial details to a high-quality, high-resolution stream architecture.

All these models encompass key aspects of segmentation design, namely skip connections (U-Net), multi-scale context (DeepLabV2, PSPNet), lightweight models (DABNet), and high-resolution feature preservation (HRNet). This choice assures good and fair comparison as they are established architectures, fully open to testing, and represent unique solutions to the key problems of segmentation of the core, which can thus offer meaningful context to show the contributions of our new IGSM-enhanced symmetric architecture. It is worth mentioning that the model proposed is not a variation in these architectures but rather a self-contained, bespoke design, the performance of which is relatively compared with these self-contained references.

## 4. Dataset

### 4.1. Dataset Description

**Figure 5:**
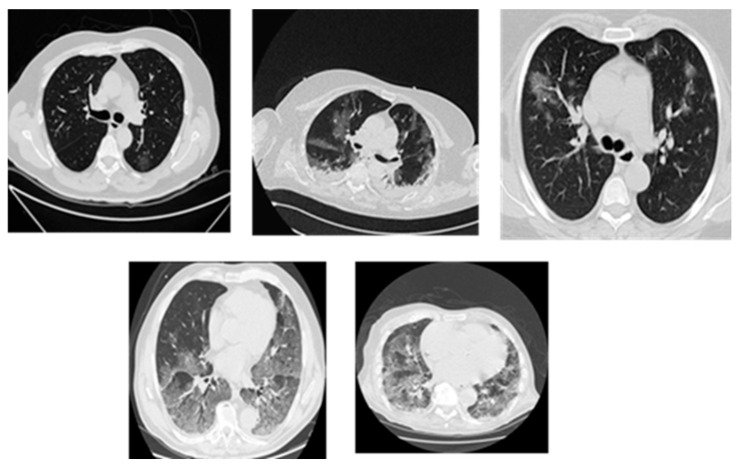
Samples of the collected dataset.

The Iraqi Lung Cancer Image Dataset (ILCID) utilized in this research was collected from three large Iraqi medical metropolitan centers, which were diverse and clinically reliable. The three hospitals are Oncology and Alternative Nuclear Medicine Hospital in Mosul (1500 images), Al-Zahrawi Special Hospital in Mosul (800 images), and Shilan Special Hospital in Duhok (700 images), whereby a total of 3000 high-resolution lung CT scan images were recorded according to ethical approval from the Iraqi Ministry of Health, Nineveh Health Directorate, Training and Human Development Center (Ethical ID Code: 2023118, approved in 7 June 2023). The collection of data started in October 2022 by obtaining formal permission from each institution. All scans were made with the Philips Brilliance barefoot CT Scanner (model 2019), where the image quality and the imaging parameters were standardized to ensure uniform recorded data. The dataset has a wide range of lung cancer manifestations, such as various tumor volumes, types, and phases of the disease (early, intermediate, and advanced). Each scan was annotated manually by expert radiologists who gave each scan accurate segmentation masks to enable powerful model training and assessments. Inclusion criteria were also very strict to make sure that it is clinically relevant and includes various types of cancer (small-cell and non-small-cell lung cancer) and diverse patient demographics to improve the generalization. Before developing the model, there was an extensive preprocessing pipeline that eliminated artifacts and low-quality scans and normalized image properties, leaving a high-quality dataset free of problems and ready to be used in advanced computer-aided diagnosis. Figure 5 shows samples of the collected dataset.

The CT scans were initially available in standard DICOM format. For this study, all images were converted to JPEG to facilitate the subsequent processing pipeline. Before network training, pixel intensity values were normalized to a fixed range. In parallel, the corresponding segmentation masks were resized to match the spatial resolution of the input images and then encoded into a two-channel one-hot representation, following the original mask convention (0 for background and 255 for lesion).

Before the segmentation model was fed with the CT scans input, a standardized preprocessing pipeline was implemented. The images and all CT slices and masks were resampled to a final constant isotropic resolution of 0.75 mm × 0.75 mm with a linear resampling protocol ensuring label integrity and a nearest neighbor resampling protocol ensuring mask integrity. The values of the Hounsfield Unit (HU) were then cropped to a window of lung values of range [−1000, 400] HU, scaled to proportion [0, 1]. A fixed size of 512 × 512 pixels was decided, and the processed slices and their corresponding masks were centered and cropped. Lastly, the segmentation masks were transformed to a one-hot coded format, which produced individual binary classes of the background (Class 00) and the nodule foreground (Class 01). This stringent and transparent curation and preprocessing process of data offers a solid base for building and assessing strong segmentation models, which could lead to effective clinical adoption.

### 4.2. Model Training and Configuration

The model training procedure commenced after finishing data preparation work. The vital parameters, including batch size and number of epochs, along with learning rate, needed to be configured. The batch size defined the number of samples that run during each training step, and the learning rate set the size of model optimization steps. For training purposes, the model received batches of 16 samples through 60 epochs, utilizing a learning rate of 0.0008. The model architecture operated from a popular segmentation framework without providing complete details about its structure. It could either use U-Net, FCN, or a combination of both. The network design featured both convolutional layers to find specific features, in addition to up-sampling layers that generated pixel-precise prediction labels. Table 1 summarizes the training hyperparameters used in this work.

The decisions about the hyperparameters were made by an empirical validation performed on a portion of the training set that was not held. The batch size of 16 was chosen because the size of 16 was the largest possible size with the limitations of the GPU memory, and it provided the same gradient estimates. It was also discovered that the learning rate of 0.0008 would achieve a stable convergence rather than instability. Training was performed with 60 epochs with an early stopping criterion tracked on the validation Dice score to ensure the model converged to reliable roots and no significant additional gains occurred afterward, avoiding unnecessary computation and subjecting the model to overfitting.

### 4.3. Evaluation Metrics for Image Segmentation

Multiple evaluative metrics are used to evaluate image segmentation models by assessing different aspects of segmentation quality performance. These evaluation metrics show how precise and thorough the model segmentation outcomes are while measuring the accuracy across all categories. Different metrics exist for evaluating segmentation models, which are frequently applied to validate such models.

Mean IoU (Intersection over Union) [41]: Among all image segmentation evaluation metrics, the Mean IoU stands out as the most popular choice. The IoU calculation evaluates how well two elements match by determining their shared area compared to their total combined areas. For each class, IoU evaluates the overlap area between the prediction and truth segmentation relative to the combined area included by the prediction and truth segmentation. A segmentation task obtains its Mean IoU through averaging all IoU values across its multiple classes. Better segmentation performance occurs when the IoU value reaches higher levels because it demonstrates an accurate fit between predicted masks and actual ones without extra inclusions. This evaluation method works well for model testing on problems containing various classes.

(7) $$mIoU=\frac{1}{N}\sum_{i=1}^{N}\frac{{TP}_{i}}{{TP}_{i}+{FP}_{i}+{FN}_{i}}$$

where *N* is the number of classes.

Dice Score [42]: The Dice Score represents an essential metric for performance evaluation of segmentation systems, while it is commonly referred to as the Dice Similarity Coefficient (DSC). This metric calculates the overlap degree between prediction and truth mask segmentation by using a formula that divides the area of shared pixels by the sum of all pixels between the two masks multiplied by two. The Dice Score presents a value between 0 and 1 that describes the extent of segmentation match. The Dice Score evaluation method shows higher overall performance when it achieves a better score because this metric demonstrates strong agreement between prediction outcomes and reference standards. Medical imaging specialists prefer the Dice Score because it successfully detects minimal discrepancies between predicted and actual masks when segmenting tumors or organs.

(8) $$DSC\left(Dice\right)=\frac{2TP}{2TP+FP+FN}$$

Accuracy [43]: The general metric Accuracy determines how well the model identifies pixels based on their classification category. The metric determines the relationship between correctly identified image pixels, including background and foreground, versus all pixels present in the image. The effectiveness of accuracy measurement becomes unreliable when datasets have extreme class distribution, including the background and the desired object. Such situations yield maximum accuracy through defaulting to the most common class outcome even when minority class segmentation is incorrect.

(9) $$Accuracy=\frac{TP+TN}{TP+TN+FP+FN}$$

Sensitivity and Specificity [44]: Sensitivity (also known as recall or true positive rate), together with Specificity (also known as true negative rate), serve as essential metrics, especially for medical applications. Model sensitivity describes how well the system detects positive instances like medical image disease regions by dividing true positives by their sum with true positives and false negatives. The detection of all positive cases requires few false negatives when the sensitivity is high in medical diagnostic scenarios. The model’s ability to correctly identify negative cases, such as healthy medical regions, determines Specificity.

It specifies the ratio of true negatives to the total number of actual negatives, which is all negative instances in the data. A model-specific output guarantees that it accurately separates normal from affected areas. In the medical domain, combining high sensitivity with specificity is crucial since it allows the model to perform efficient detection of positive cases as well as minimize false positives, which might lead to some useless treatment or other diagnostic measurements.

(10) $$Sensitivity=\frac{TP}{TP+FN}$$

(11) $$Specificity=\frac{TN}{TN+FP}$$

### 4.4. Baseline Results

For early comparison, the performance simulations are computed by analyzing a controlled baseline to isolate the effect of the Improved Group–Shuffle Module (IGSM) by first performing analysis on a variety of symmetric encoder–decoder versions recurrently constructed using our architecture, all trained under the same circumstances. These baselines have the same core symmetric form, yet they vary in terms of internal feature processing mechanisms. Table 2 shows a comparative performance study of these in-house designed models, which is an internal ablation study before external benchmarking.

**Figure 6:**
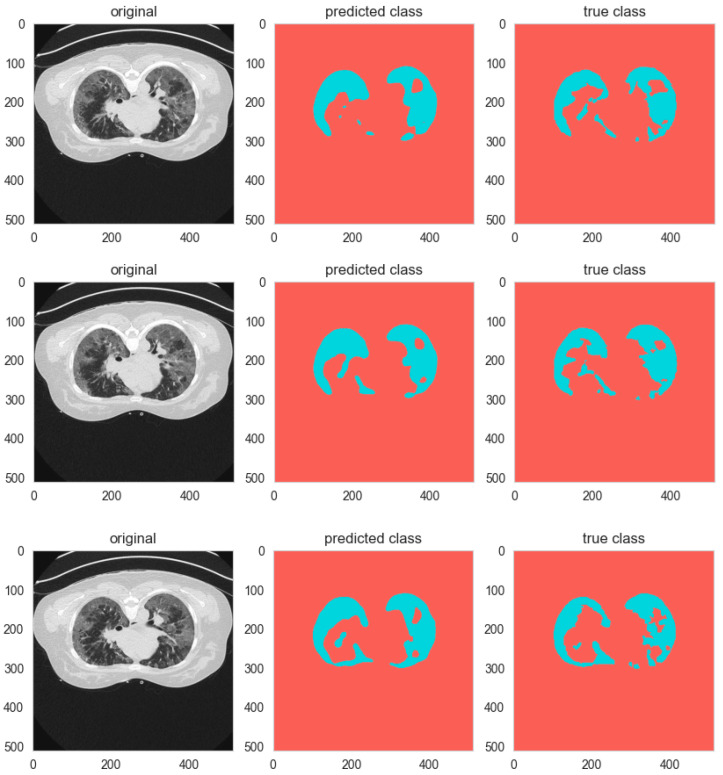
Samples of segmentation outputs of the baseline models.

The Baseline (Standard Convolution) model uses an otherwise convolutional symmetric encoder–decoder, with no special or grouping mechanisms, and demonstrated a Mean IoU of 0.7241 and a Dice Score of 0.9383. The addition of isolated modules—Channel or Spatial—produced moderate improvements on some measures, but often at the expense of others, including Specificity in the Channel measure. A better balance was gained when both were integrated into a Dual baseline with a Mean IoU of 0.7468, as indicated in Figure 6.

It is important to note that we added our Improved Group–Shuffle Module (IGSM) into the identical symmetric backbone, and this led to the best performance in all aspects. This suggested configuration of Baseline + IGSM obtained a Mean IoU of 0.7735 and a Dice Score of 0.9665, which showed clear and significant improvements over scenarios. This ablation ascertains that the mechanism of the IGSM of grouped feature transformation and cross-group shuffling is better at increasing feature diversity and discriminative ability in lung nodule segmentation than traditional mechanisms. These low-level comparisons confirm that the IGSM added to the performance of our entire model prior to external benchmarking with set architectures.

### 4.5. Group and Shuffle Results

Table 3 demonstrates the performance assessment of three configurations for Group–Shuffle, consisting of two groups with Channel-wise shuffle, four groups with Channel-wise shuffle, and eight groups with Spatial-wise shuffle. The evaluation of different models used Mean IoU and IoU per Class (Class 00 and Class 01), Accuracy, Dice Score, IoU Score, Sensitivity, and Specificity metrics with a 512 × 512 image size for performance assessment. The experimental results demonstrate that both the group quantity and shuffle approach determine the model’s segmentation output.

The experimental data demonstrate that Mean IoU improves steadily as the model adds more groups. Increased segmentation performance occurs when using a 2-group Channel-wise shuffle since it reaches a Mean IoU of 0.721, although additional groups would yield better results. Segmentation performance improves significantly because the 4-group Channel-wise shuffle configuration reaches a Mean IoU value of 0.7453. The highest accuracy for segmentation occurs with the 8-group Spatial-wise shuffle since it results in a Mean IoU score of 0.7735. The model demonstrates an enhanced capability to properly identify both foreground and background elements when the shuffle type changes from Channel-wise to Spatial-wise while increasing the number of groups.

The model displays a constant, powerful performance in detecting Class 00 (background) based on the IoU measurements throughout testing. The 2-group Channel-wise shuffle model produces a segmenting performance of 0.995 for background identification. Using a 4-group Channel-wise shuffle enables a minimal increase in Class 00 IoU up to 0.996, and the 8-group Spatial-wise shuffle model reaches its highest performance of 0.9973 with Class 00 segmentation, thus demonstrating improved background segmentation capability through group number growth and Spatial-wise shuffle application.

IoU Class 01 (foreground) has a staggeringly high increase with increasing group numbers. Only using a 2-group Channel-wise shuffle for foreground segmentation results in 0.447 IoU. The 4-group Channel-wise shuffle method raises foreground segmentation to 0.495, and the 8-group Spatial-wise shuffle increases to 0.5543 for a better result. The most powerful augmentation for foreground segmentation is the Group–Shuffle technique, which receives favorable results by utilizing multiple groups with Spatial-wise shuffle types. This advancement is an important one for medical image segmentation since it requires a very high accuracy in foreground object detection.

The accuracy of the model improves as we introduce more groups in its setup. Its accuracy achieves 0.981 under a Channel-wise shuffle with a group number of 2, and increases to 0.984 with group number being set to 4 for Channel-wise shuffle, and finally reaches 0.9873 by using the Spatial-wise shuffle with a group number equaling 8. With more sophisticated grouping strategies and improved shuffle types, the model becomes increasingly able to decide between the cells the background pixels.

Generally, the Dice Score metric calculates how well the predicted masks fit the real segmentation masks. The results indicate that the 2-group Channel-wise shuffle achieves a score of 0.92, the 4-group Channel-wise shuffle obtains a score of 0.94, and benefits profusely from our method to achieve the highest at about 0.9665 for the 8-group Spatial-wise shuffle. The high sensitivity of Dice Score toward accurate foreground segmentations suggests that such score gains are indicative of improved quality of the segmentation, particularly with respect to foreground structures.

We can see that the same happens when it comes to the IOU Score, which indicates the overall segmentation performance across both of the classes. The 2-group Channel-wise shuffle has an IoU Score of 0.87, the 4-group Channel-wise shuffle achieves an IoU score of 0.89, and the 8-group Spatial-wise shuffle achieves an IoU score of 0.9203. This demonstrates that the use of Spatial-wise shuffle and a greater number of groups is favorable for overall segmentation performance.

**Figure 7:**
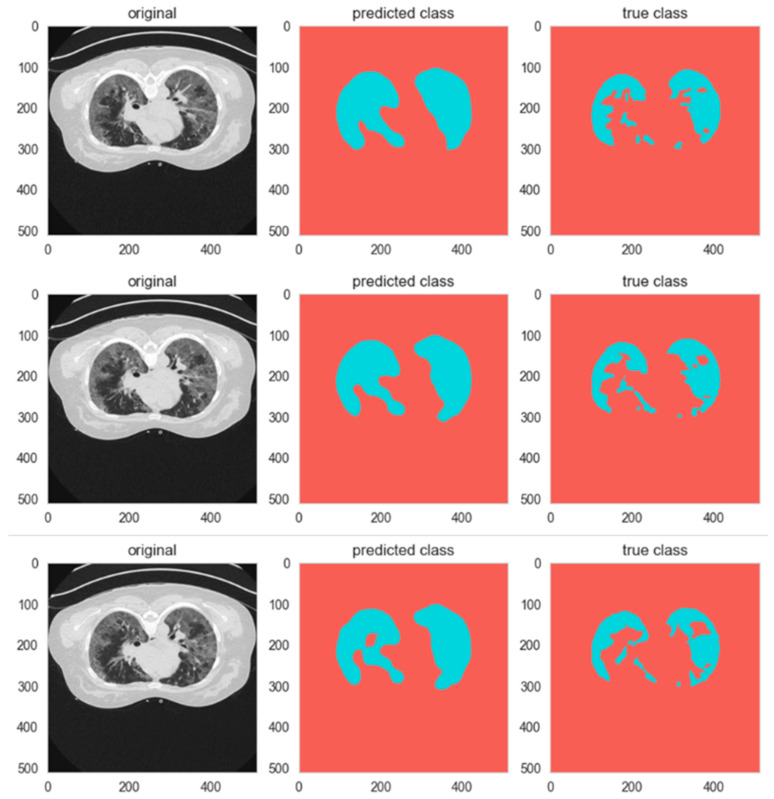
Sample of group and shuffle segmentation outputs.

The analysis is worth deeper investigation; Specificity, also called the true negative rate, assesses the performance of the model to identify background pixels, while Sensitivity, also known as the true positive rate, measures its ability to identify foreground objects. Both measures improve as the number of groups increases. For the 2-group Channel-wise shuffle setup, Sensitivity and Specificity are at 0.963 and 0.94, respectively. When we move to the 4-group Channel-wise shuffle, the values are 0.97 and 0.945. However, the top one among these is the 8-group Spatial-wise shuffle model, which has a Sensitivity of 0.9757 and Specificity of 0.9532. These higher values indicate the manner in which the use of additional groups and the Spatial-wise shuffle technique assist the model in segmenting foreground objects and categorizing background pixels more effectively. Figure 7 shows samples of Group and Shuffle results.

### 4.6. Optimal Configuration of Group Shuffle + CNN-Based Encoder–Decoder

Several sets of experiments were conducted to investigate the different group convolution settings and batch sizes in the proposed model. Among all these experiments, a batch size of 4, Group Convolution 4, 7 = 16, and Group Convolution 5, 6 = 2 result in the best overall performance. This configuration strikes the best balance between segmentation accuracy, foreground and background discrimination, and computational efficiency. The Mean IoU, Dice Score, Accuracy, Sensitivity, and Specificity of the resulting model show the effectiveness of incorporating group convolutions along with Channel-wise and spatial shuffle into enhancing the baseline CNN-based encoder–decoder model. Table 4 summarizes the key metrics for this optimal configuration.

### 4.7. Benchmarking and Comparative Analysis

A key conclusion of benchmarking segmentation models is the trade-off between performance (accuracy, computational efficiency, and structural fidelity) and structural fidelity. Table 5 contains the benchmarking findings, where the performance of a CNN-based encoder–decoder baseline is compared to that of the proposed Group–Shuffle-enhanced version (G&S+), along with a number of other configurations. The analysis is based on popular measures of segmentation, such as Mean IoU, Accuracy, Dice Score, Sensitivity, and Specificity. The G&S+ CNN-based encoder–decoder model is the most successful as it attains the highest results in all metrics with Mean IoU = 0.773, Accuracy = 0.9873, Dice = 0.9665, Sensitivity = 0.9757, and Specificity = 0.9532, indicating obvious effects of background and foreground segmentation.

In the case of the CNN-based encoder–decoder, the background segmentation (Class 00 IoU = 0.995) is excellent, as the size of the background areas and their ease of recognition are very large. However, it has trouble with the foreground splitting, giving a low, insignificant overlap (IoU) value of 0.453 at Class 01, meaning that it is not very effective at the small, irregular nodules. This can be overcome by including a Group–Shuffle mechanism. The G&S+ model is able to not only maintain high background performance (Class 00 IoU = 0.997), but also increase foreground IoU to 0.554. This is due to increased feature variability and cross-group exchange of the information that allows the model to be more sensitive to detect fine and low-contrast structures like lung nodules.

**Figure 8:**
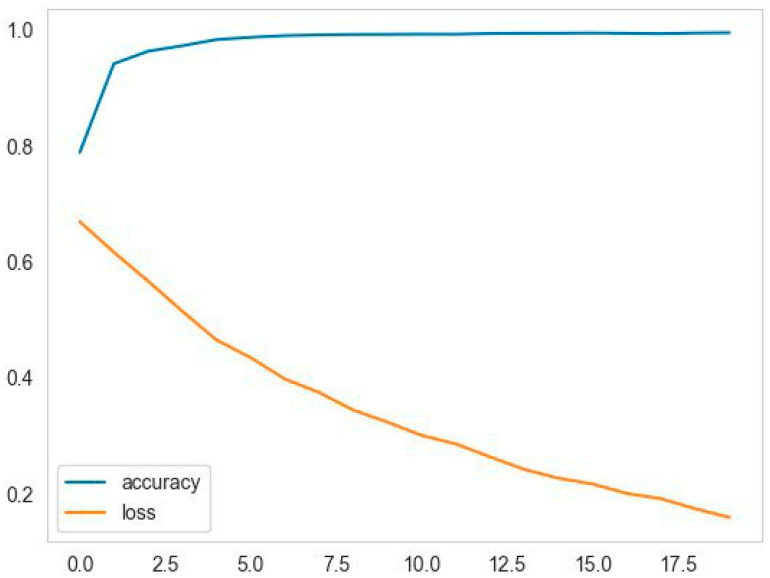
The dynamics training of the suggested G&S+ model.

In addition to the social performance of classes, G&S+ increases the quality of the segmentation in the whole picture, with Mean IoU increasing to 0.773, whereas the whole-segmentation Accuracy increases to 0.9873. The Dice Score increases to 0.9665 compared to 0.9383, which implies more accurate predictions and ground-truth masks. The Sensitivity is also improved to 0.9757, which indicates the better capability of the improved model to pick minute foreground objects that are very important in medical imaging. Specificity increases to 0.9532, indicating a smaller level of false positives. Figure 8 gives sample predictions in the baseline and G&S+ models.

In order to put the benchmarking findings into perspective, it is imperative to compare the CNN-based encoder–decoder as well as the proposed Group and Shuffle architecture with some of the most popular segmentation models, such as U-Net, DeepLab, PSPNet, HRNet, and DABNet. These architectures embody varied design ideologies and are tuned to various imaging directions, computational limitations, and segmentation specifications.

U-Net has been widely used in the past in medical image segmentation because of its symmetric encoder–decoder architecture as well as its large amount of skip connections, which effectively retain the spatial information and allow its use to give accurate predictions down to the pixel level. Although the U-Net is highly successful in other fine anatomical boundaries, its large dependence on a large feature map size leads to increased memory consumption and low performance in a large-scale or real-time setting.

DeepLab V1/V2 introduces atrous (dilated) convolutions, which allow the model to learn to use multi-scale contextual information without the use of aggressive down-sampling. It is very successful on the problematic cases of natural scenes where there are objects that appear in different sizes. Nevertheless, dense multi-scale feature extraction involves high computational costs, which restrict the applicability of the model in resource-constrained systems.

PSPNet improves contextual perception by pooling boards of a space pyramid that ensures that the network merges global and local features. The quality of segmentation through this capability is superior in large-scale data, especially in scene parsing and satellite data. Although it is accurate, PSPNet is computationally expensive, and training the model takes a significant amount of time and requires a lot of GPU memory, which limits its applicability in real-time use.

HRNet preserves high resolutions of feature representations of the entire network, which makes it possible to recover fine boundaries and structural details. This renders the use of HRNet highly useful in operations like high-resolution medical imaging, facial parsing, and aerial analysis. The main weakness of HRNet is that it has critically large computation and memory requirements, owing to the presence of multi-resolution branches used in parallel.

DABNet uses two forms of shuffling to highlight salient features in the feature space, including channel and spatial, and is therefore better at discrimination in noisy or complicated environments. Although mechanisms enhance the model’s concentration on significant structures, they introduce more architectural complexity and could slow down the inference process, particularly on low-power systems.

On the whole, the models, shown in Table 6, offer a variety of strengths and have trade-offs between accuracy, computational efficiency, and context awareness. The point of this comparison is that it is necessary to create lightweight and robust architectures, encouraging the addition of Group–Shuffle modules to the CNN-based encoder–decoder to obtain a more balanced accuracy–efficiency tradeoff.

### 4.8. Computational Efficiency and Training Dynamics Analysis

Effective use in a practical, clinical work environment demands that models are not only precise but also efficient in computation. A comparative analysis of the proposed G&S+ model and the benchmark architectures is performed in a full computational manner in Table 7. The experiments were operated on the same hardware (NVIDIA RTX 4090 GPUs with 24GB VRAM) and software system (TensorFlow 2.07).

The architecture proposed shows a perfect balance between performance and efficiency. It is also much faster than heavier networks, such as PSPNet and HRNet, since its training time per epoch matches that of U-Net and DABNet. Importantly, its inference time of only 17 ms per image (practically, 59 FPS) is the lowest of all the compared models, which indicates its appropriacy and use in real-time clinical practice. The model has a parameter efficiency of 25.3 million parameters as well, which is lower compared to most benchmarks, and lowers the memory cost of deployment.

### 4.9. Training Dynamics Analysis

In order to give a complete understanding of the learning process, computational performance, and efficiency in the implementation issues, this section gives a detailed analysis of model training, efficiency, and measures of reproducibility. The dynamics training of the proposed G&S+ model during the training is demonstrated in Figure 8, and Table 7 shows a detailed comparison between the computation of the proposed architecture and the benchmark architectures.

As shown in Figure 8, the training accuracy improves gradually across epochs and reaches a stable plateau, whereas the training loss decreases consistently and converges smoothly. This behavior indicates that the optimization progressed stably during training.

### 4.10. Clinical Translation and Practical Deployment Considerations

The transition of AI models for lung nodule segmentation into practical clinical environments requires much more than just their performance scores. From the aspect of integrating radiological workflows, the fast inference time of the model at approximately 17 ms per image or around 59 FPS will enable near real-time processing in current Picture Archiving and Communication Systems (PACS) platforms. For instance, based on the normal range of slices for a chest CT examination, which is in the range of 200 to 300 slices, automatic segmentation overlays can be produced in around 3 to 5 s so that the results can be immediately verified by radiologists without any delays in reporting.

Regarding clinical heterogeneity, the scope of lung nodules in terms of size, opacities (solid, part-solid, and ground-glass opacities), and location can vary greatly. Experiments on the ILCID, which include all possible heterogeneities like these, have shown that models can perform well on different cases. The group–shuffle component of the IGSM works very well in handling different morphological heterogeneities of images and thus results in higher foreground segmentation accuracy (IoU of 0.5543 on Class 01). This can lead to fewer cases of false negatives of ground-glass opacities.

However, it also aligns well with major clinical objectives. Its high Sensitivity of 0.9757 enables it to be useful as a first reader in the screening process, where it can indicate potential areas of disease, which then require verification by the radiologists. However, its Specificity of 0.9532 suggests that it still has some false positives, which can be improved by taking into account the clinical characteristics of the patient, such as age or smoking status. In addition, its capability to perform precise segmentation allows it to perform precise volume estimation, which can be useful in treatment evaluation according to RECIST criteria.

There still exist several issues in deploying this model, which are being worked upon. Domain adaptation can be an issue when this model has to be deployed in different populations, machines, and protocols, especially because the training data has been obtained from Iraqi patient populations. Then, to ensure clinical integration, DICOM-compliant interfaces, safe handling of patient data, and adherence to HIPAA or GDPR will be required. At the same time, to increase radiologist acceptance, uncertainty estimation and visualization will be explored.

The clinical impact of this proposed system is substantial. This proposed system can serve as a deciding tool for deciding areas that need further analysis in under-resourced hospitals, reducing the shortage of trained radiologists. It can help alleviate radiologist burnout by reducing time-consuming segmentation in high-volume hospitals, leaving more time for higher-level reasoning for diagnosis. It is important to note that it is possible to implement this system on a standard working computer due to its moderate parameter complexity (25.3 M parameters) and computational complexity. Prior to its clinical utilization, additional validation work would be important, such as multi-institutional prospective analyses and comparisons of reader performance with and without the assistance of AI. In aggregate, the IGSM-assisted architecture offers significant promise for efficient, accurate, and viable automated segmentation of the lung nodule within the context of the chest CT scan.

## 5. Conclusions

The fusion of CNN-based encoder–decoder and Group–Shuffle blocks into one model structure improves the CT image lung cancer segmentation effectiveness significantly. It can be seen that a significant performance increment is achieved in foreground segmentation by this model, as its performance has a crucial effect on the detection accuracy of lung nodules. It achieves better segmentation results with efficient computation by embedding Group–Shuffle into the CNN-based encoder–decoder model. The hybrid method has promising capability to meet the requirements of automated lung cancer diagnosis, as it is shown to perform well in complex clinical settings with high accuracy. The experimental results indicate that Group–Shuffle methods can improve the capacity of segmentation models to adjust to clinical medical imaging tasks. The improved model is a diagnostic method used by radiologists to identify primary lung cancer cases early so as to enhance the accuracy of the diagnosis. Further studies to review and incorporate CT scans with other imaging, including PET or MRI, to improve identification and segmentation of nodules may be required. Also, it would be beneficial to optimize the model to be deployed in real-time on an edge device or at a clinical workstation to perform immediate analysis in a practical environment. Lastly, the use of semi-supervised or self-supervised learning methods may enhance the model to generalize to a wide and limited set of data.

Besides providing high results on the ILCID, some limitations should be considered. To begin with, it is expected that the appraisal based on one curated dataset requires prudence over the extrapolation to other populations and imaging standards. The next phase of work will be concerned with the multi-center validation based on different geographically distributed datasets. Second, the existing implementation is based on the accuracy of segmentation but does not include malignancy prediction, which would be a promising avenue to integrate segmentation with classification in an end-to-end model. Third, although computational efficiency is shown, more optimization would need to be performed prior to real, clinical deployment, implementation with individual PACSs, and being compatible with the IT infrastructure of hospitals. In spite of these restrictions, the presented architecture demonstrates the promising features of clinical translation, especially its accuracy (0.9665 Dice Score), speed (17 ms inference), and the efficiency of its parameters (25.3 M parameters).

## Figures and Tables

**Table 1:** Training hyperparameters.

Parameter	Value	Description
Batch size	16	The number of images processed in each batch.
Epochs	60	The total number of times the model will iterate over the training dataset.
Learning rate	0.0008	The rate at which the model updates its weights during training.

**Table 2:** Segmentation performance of the baseline models.

Model Configuration	Mean IoU	Dice Score	Accuracy	Sensitivity	Specificity
Baseline (Standard Convolution)	0.7241	0.9383	0.9796	0.9541	0.9233
Baseline + Channel	0.7391	0.9246	0.9756	0.9307	0.8762
Baseline + Spatial	0.7315	0.9312	0.9778	0.9432	0.9014
Baseline + Dual (Channel + Spatial)	0.7468	0.9421	0.981	0.9589	0.9347
Baseline + IGSM (Proposed)	0.7735	0.9665	0.9873	0.9757	0.9532

**Table 3:** Group and shuffle performance results.

Number of Groups	Shuffle Type	Mean IoU	Accuracy	Dice Score	IOU Score	Sensitivity	Specificity
2	Channel-wise	0.721	0.981	0.92	0.87	0.963	0.94
4	Channel-wise	0.7453	0.984	0.94	0.89	0.97	0.945
8	Spatial-wise	0.7735	0.9873	0.9665	0.9203	0.9757	0.9532

**Table 4:** The best Group–Shuffle + CNN-based encoder–decoder segmentation performance results.

Performance Metric	Result
Mean IoU	0.7589
Dice Score	0.9284
Accuracy	0.9768
Sensitivity	0.9349
Specificity	0.8842

**Table 5:** Benchmarking results.

Model	Mean IoU	Accuracy	Dice	Sensitivity	Specificity
Benchmark	0.724	0.9796	0.9383	0.9541	0.9233
enhanced Group–Shuffle model	0.773	0.9873	0.9665	0.9757	0.9532
Alternative configurations	0.7391	0.9756	0.9246	0.9307	0.8762

**Table 6:** Summary of segmentation model characteristics.

Model	Key Architecture	Core Advantage	Computational Demand
U-Net	Symmetric encoder–decoder + skip connections	Preserves fine spatial details	Medium
DeepLab V1/V2	Atrous convolutions	Multi-scale context extraction	Medium
PSPNet	Spatial pyramid pooling	Integrates global and local context	High
HRNet	Parallel high-resolution streams	Maintains high-resolution features	High
DABNet	Dual	Saliency-focused feature extraction	Medium
Proposed G&S+ CNN-based encoder–decoder	Grouped conv + feature shuffle	High feature diversity + low computational cost	Medium-low

**Table 7:** Computational performance comparison.

Model	Avg. Training Time/Epoch (s)	Avg. Inference Time/Image (ms)	Parameters (M)
U-Net	142	18	31
DeepLabV2	167	22	44
PSPNet	215	29	65.7
HRNet	298	35	63.6
DABNet	151	19	7.6
Proposed G&S+	148	17	25.3

## Data Availability

The original data will be made available upon request from the authors.
